# Exploration of bioflocculant production of endophytic bacterium: assessment of characteristics and efficiency of its bioflocculant in wastewater treatment

**DOI:** 10.1007/s11356-026-37543-z

**Published:** 2026-03-03

**Authors:** Getrude Mothootsile Ramonyai, Tlou Nelson Selepe, Tsolanku Sidney Maliehe, Nkoana Ishmael Mongalo, Cyril Tlou Selepe, Kgabo Moganedi

**Affiliations:** 1 Department of Biochemistry, Microbiology and Biotechnology, University of Limpopo, Private Bag X1106, Polokwane, 0727 South Africa; 2 Department of Water and Sanitation, University of Limpopo, Private Bag X1106, Polokwane, 0727 South Africa; 3 Department of Biochemistry, Genetic and Microbiology, University of KwaZulu Natal, Private Bag X 54001, Durban, 4000 South Africa; 4 UNISA College of Agriculture and Environmental Sciences, University of South Africa, Private bag X06, Florida, 0710 South Africa; 5 Department of Chemical Sciences, University of Johannesburg, Private Bag X X61, Johannesburg, 2006 South Africa

**Keywords:** *Adansonia digitata*, Bioflocculant, *Bacillus albus*, Biosynthetic gene clusters, Characterisation, Removal efficiency

## Abstract

Endophytic bacteria are promising sources of potent bioflocculants, yet their diversity, biosynthetic potential and utility remain underexplored. This study focused on isolating bioflocculant-producing endophytes from *Adansonia digitata*, exploration of biosynthetic gene clusters and application of its bioflocculant in wastewater treatment. The standard method and 16S rRNA analysis were used for isolation and identification of endophytic bacteria. The polymerase chain reaction was employed to detect the presence of Type I Polyketide Synthase (PKS-I KS), Type II polyketide synthase and non-ribosomal peptide synthetase gene clusters. The infrared spectrometry, energy-dispersive X-ray and X-ray diffraction analysis were employed for identification of functional units, elements and crystallinity. Its efficiency was evaluated in wastewater treatment using the Jar test. Amongst the 18 isolates, Isolate Gt18, which was identified as *Bacillus albus,* had the highest bioflocculant production with flocculating activity of 99%. It revealed the presence of PKS-II. Its bioflocculant composed of hydroxyl, amine and carbonyl functional groups. It had carbon (1.48%), oxygen (40.47%), phosphate (17.44%) and potassium (28.58%). It revealed crystalline nature. It reduced chemical oxygen demand and turbidity by 70 and 99.8% for sewage wastewater and 57 and 75% for brewery wastewater, respectively. The bioflocculant from *B. albus* illustrated high potential applicability in wastewater treatment*.*

## Introduction

Microbial bioflocculants are extracellular polymers (EPS) secreted by bacteria, algae and fungi during substrate metabolism, growth phase, cell lysis and degradation of chemical components (Pu et al. 2020). Amongst the identified microbial flocculant producers, bacterial strains are the most common producers, owing to their high metabolic capabilities for producing bioflocculants (Chen et al. 2017). Bioflocculants are made up of proteins, lipids, glycoproteins, polysaccharides and nucleic acids (Alias et al. 2022). As compared to their chemical counterparts, they are more environmentally benign and innocuous to humans (Okaiyeto et al. 2020). According to Yang et al. (2025), although bioflocculants have many advantages, their industrial applications in wastewater treatment have been constrained by poor yields and low efficiencies, which often translate into high production costs.

The drawbacks of low bioflocculant yield have prompted the exploration of untapped environments in search of high bioflocculant-producing bacteria with enhanced bioflocculant efficiencies. The rhizosphere is perceived to be a habitat rich in a diversity of microorganisms, including bacteria (Hakim et al. 2021). The bacteria are mostly found on the surface of plants (epiphytes) as well as in the interior of the plant tissues (endophytes) (Costa et al. 2012). Endophytes reside within the host tissue without harming the health and functionality of the host plant (Hardoim et al. 2008). Lodewyckx et al. (2002) and Singh et al. (2022) indicated that bacterial endophytes secrete various types of secondary metabolites, which can be applied in a variety of industries, including environmental contamination control such as water bioremediation (Manganyi et al. 2024). However, research on the use of endophytes in environmental science, particularly in the production of bioflocculants, is still limited.

Most studies tackle the challenge of low bioflocculant yields mainly through the identification of potent bioflocculant producers and optimisation of their culture conditions. Although these strategies do improve bioflocculant yields to some extent, bioflocculant production is mainly influenced by the expressed flocculation-related biosynthetic gene clusters and genes (Salehizadeh et al. 2018). Based on literature, many non-flocculating extracellular functional genes have been reported. However, there are scant reports on the biosynthesis genes of bioflocculant producers (Qi et al. 2019; Fu et al. 2020). The exploration of genes expressed during bioflocculant production can ease the engineering of high bioflocculant-producing strains, consequently overcoming the uncertainties in reproducing these biopolymers (Fu et al. 2020).

The majority of endophytic bacterial research, according to Etminani and Harighi (2018), focuses on agricultural plants. However, there is a lack of studies on the function, diversity and interactions of endophytic bacteria associated with forest trees and plants, with *Adansonia digitata* not being the exception. Literature has reported on endophytes from *A. digitata* as potential reservoirs of bioactive compounds possessing medicinal attributes (Kumar et al. 2023); however, there is a lack of research, particularly in bioflocculant production for water and wastewater treatment. Hence, we isolated an endophytic bacterium with high bioflocculant production from the roots of *A. digitata* and explored its biosynthetic genes responsible for bioflocculant production.

The characterisation of bioflocculants is done through advanced techniques to better understand their physical, chemical, thermal and structural properties (Rajivgandhi et al. 2021). These various techniques include Fourier transform infrared spectroscopy (FTIR), scanning electron microscopy (SEM) and X-ray diffraction (XRD) (Srinivasan et al. 2023). FTIR is used to determine the functional groups such as carbonyl, hydroxyl, amine and phosphate groups in bioflocculants by producing an infrared absorption spectrum (Botirova et al. 2023). SEM is an electron microscope that uses a high-energy beam of electrons to divulge the surface morphology (shape and size) of bioflocculants (Kumar et al. 2023; Roy and Mohanty 2020). XRD is a conventional technique employed to determine the lattice structure of a crystalline substance. Moreover, it provides cell dimensions and bond angles of bioflocculants (Roy and Mohanty 2020). These methods were used in this study to characterise the produced bioflocculant.

Therefore, in this study, we aimed to isolate and identify endophytic bacterium from the roots of *A. digitata*. We further identified the functional flocculation-related biosynthetic gene clusters to better understand the biological processes that underpin bioflocculation production. Lastly, we characterised the extracted bioflocculant in terms of its physical–chemical properties.

## Materials and methods

### Sample collection

**Fig. 1:**
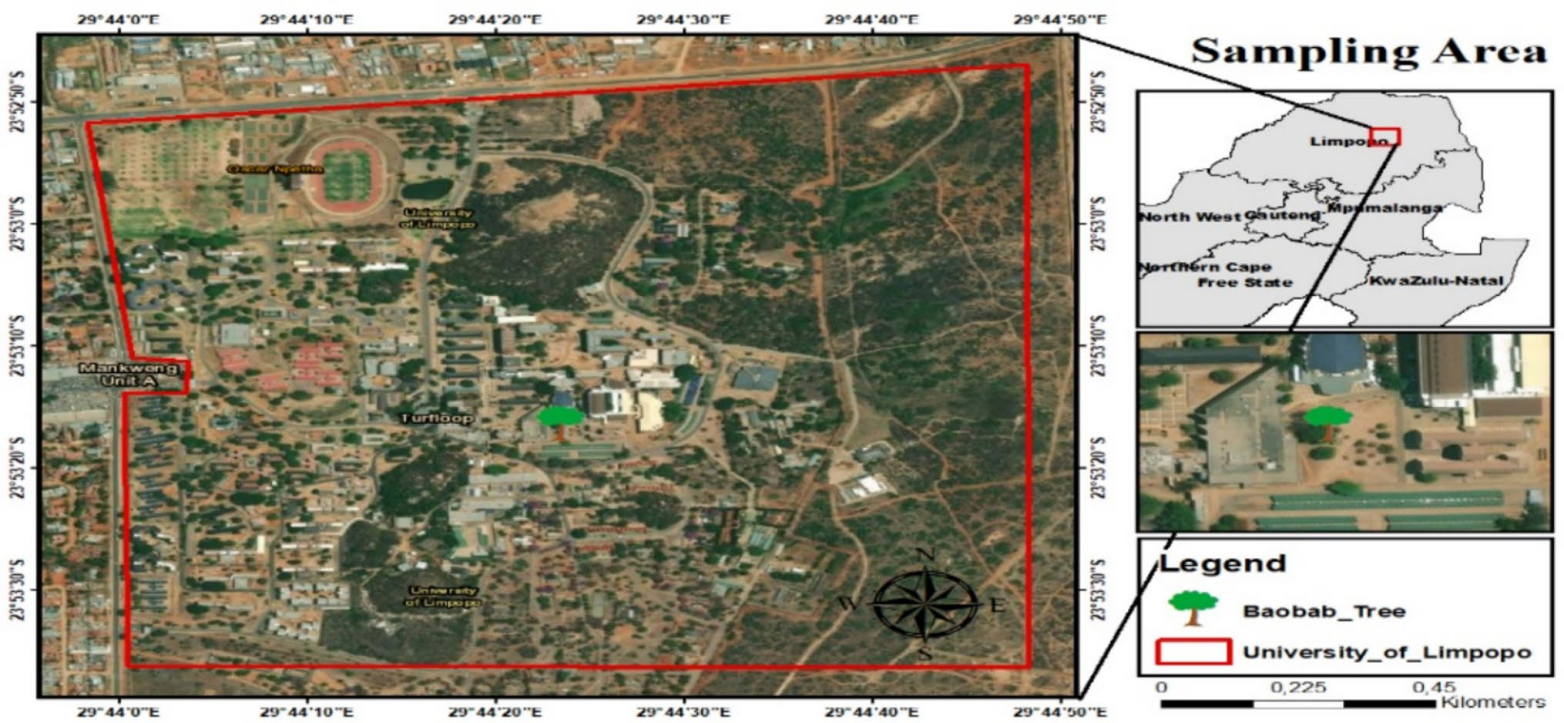
Map of the sampling location of *A. digitata*

The roots of *A. digitata* were obtained from the University of Limpopo grounds (23°53′20″ S, 29°44′20″ E) (Fig. 1). The roots were cut into 10 to 15 cm pieces, then packaged into sterile lockable plastic bags and transported to the microbiology laboratory, where the isolation procedure was performed immediately upon arrival.

#### Surface sterilisation of the roots

The roots of *A. digitata* were washed thoroughly under running tap water to remove any dirt and debris and then air dried. Afterwards, the surfaces of the roots were disinfected by dipping them in 75% ethanol for 2 min, followed by dipping in 5% sodium hypochlorite solution for another 2 min to remove epiphytes. They were then rinsed six times in sterile deionised water to remove any last traces of the chemical sterilants. To confirm the disinfection process, aliquots of the sterile water used in the final rinse were plated onto the bioflocculation production medium (BPM), which was composed of constituents listed in Table 1. The medium was supplemented with 1 mL/L of funginex antifungal agent. Subsequently, the inoculated agar plates were incubated at 30 °C for 7 days and observed for bacterial growth (Nxumalo et al. 2020).

**Table 1:** Agar medium composition for isolating and screening microorganisms for bioflocculant production

Ingredients	Amount
Glucose	20.0 g
Yeast extract powder	0.5 g
Urea	0.5 g
(NH_4_)_2_SO_4_	0.2 g
K_2_HPO_4_	5.0 g
KH_2_PO_4_	2.0 g
NaCl	0.1 g
MgSO_4_.7H_2_O	0.2 g
Agar	15 g
Autoclaved distilled water	1 L

#### Isolation of endophytic bacteria

Isolation of endophytic bacteria was performed using the method by Khumalo et al. (2025), whereby a sterile mortar and pestle were used to crush the disinfected roots in 50 mL of sterile 0.85% saline solution to release the endophytes. To reduce the microbial concentration, the root extract was serially diluted in a sterile 0.85% saline solution. About 100 µL of each diluted (10^–1^, 10^–2^ and 10^–3^) and undiluted sample were pipetted and spread-plated on the antifungal supplemented bioflocculation production media (BPM) agar plates using a sterile glass spreader. The plates were incubated at 30 °C for 7 days. After that, colonies were selected at random based on variations in morphology, size and colour. The selected isolates were purified twice on nutrient agar prior to the screening for bioflocculant production.

#### Screening for bioflocculant-producing bacteria

Pure bacterial isolates were inoculated into 250 mL conical flasks containing 50 mL sterile BPM broth. The bacteria were then incubated in a shaker (Lasec Being Incubator, BSI-21) at 30 °C for 3 days. Afterwards, the culture broth was centrifuged at 8000 rpm for 15 min to remove the bacterial cells. The cell-free supernatant was used to measure the flocculating activity. A non-flocculating strain, *Escherichia coli* (ATCC 10536), was included as a negative control (Chen et al. 2017).

#### Determination of bioflocculant activity

To ascertain the flocculating activity (FA) of the bioflocculant from the cell-free supernatant, the technique outlined by Maliehe (2018) was applied. Briefly, 2 mL of the cell-free supernatant was pipetted into 95 mL of the 0.3% kaolin clay suspension and 3 mL of 1% CaCl_2_. The mixture was vigorously agitated at 200 rpm for 60 s to reduce repulsion forces between particles and was gently shaken at 40 rpm for 2 min to promote agglomeration. The mixture was then poured into a 100 mL measuring cylinder and allowed to stand for 5 min. The control was done similarly, except that the cell-free supernatant was replaced by 2 mL of deionised water. A UV/Vis spectrophotometer (Jenway, 7205) was used to measure the optical density (OD) of the transparent upper phase layer at 550 nm after 2 mL of the layer had been aseptically extracted. The percentage flocculation activity (%FA) was calculated using the formula below:

1 $${\%FA}=\frac{{A}_{0}-{A}_{1}}{{A}_{0}}\times 100$$

whereby *A*_0_ and *A*_1_ are the ODs of the control and sample, respectively. The isolate that gave the highest bioflocculant production, expressed as FA (> 50%) was selected for identification and further studies.

#### Phenotypic identification and biochemical properties of the selected bioflocculant producers

The chosen bacterial isolate was phenotypically characterised by the Gram staining technique. Bacterial smears were prepared by heat-fixing on glass slides. The slide was placed on a tray and the smears flooded with crystal violet for 1–2 min, then rinsed with water. The smear was further soaked with Gram’s iodine for 1 min and rinsed with water. Subsequently, the smear was rinsed with 95% ethanol for 10–15 s and rinsed again with water followed by soaking the smear with counter stain safranin for 1 min and washed thoroughly with water then air dried. The findings were examined with a compound Brightfield microscope (Leica, DM500) while submerged in oil. Furthermore, the biochemical properties (spore, citrate, indole, oxidase, Voges-Proskauer, nitrate reduction, catalase, and fermentation tests) of the selected isolate were determined.

#### Molecular identification of bioflocculant-producing bacteria

The bioflocculant-producing bacterial isolate was identified by 16S rRNA sequencing, outsourced to Inqaba Biotechnical Industries (Pty) Ltd., South Africa. Genomic DNA was extracted using the Quick-DNA™ Fungal/Bacterial Miniprep Kit (Zymo Research, D6005). The DNA purity and concentration were determined using a Thermo Scientific nanodrop OneC, Microvolume UV/Vis spectrophotometer. PCR amplification of the 16S rRNA gene was performed using universal primers 27 F (5′–AGAGTTTGATCMTGGCTCAG–3′) and 1492R (5′–CGGTTACCTTGTTACGACTT–3′), with NEB One Taq 2X Master Mix. The thermal cycling conditions included initial denaturation at 94 °C for 5 min, followed by 35 cycles (94 °C for 30 s, 50 °C for 30 s, 68 °C for 1 min), and a final extension at 68 °C for 10 min. PCR products were visualised on a 1% agarose gel stained with EZ-vision® dye, and NEB Fast Ladder (N3238) was used as a size marker. Amplicons were purified (Zymo, D4050) and sequenced bidirectionally (Nimagen, BRD3-100/1000) using an ABI3730xl Genetic Analyzer. Chromatograms were viewed with Finch TV, and sequences assembled using CLC Bio Main Workbench. Identification was done via BLASTn on NCBI, with ≥ 99% query coverage and identity (Chigede et al. 2024).

#### Biosynthetic gene clusters responsible for bioflocculant production

The extracted DNA was also used to identify the biosynthetic gene clusters responsible for bioflocculant production. Briefly, a volume of 25 µL, consisting of 12.5 µL of NEB OneTaq 2X Master mix with a standard buffer, 2 × 1 µL of forward and reverse primers, 2 µL of the DNA sample (10–50 ng/mL) and 8.5 µL of nuclease-free water, was prepared. The following primers were used to amplify the extracted DNA: KSF (5′–GTSCCSGTSSCRTGSSHYTCSA–3′) and KSR (5′-TSGCSTGCTTGGAYGCSATC-3′) targeting PKS-I KS and methyl malonyl transferase domains; KsaF (5′–TSGCSTGCTTGGAYGCSATC–3′) and KsaR (5′–TGGAANCCGCCGAABCCGCT–3′) targeting PKS-II KSa genes; A3F (5′-GCSTACSYSATSTACACSTCSGG-3′) and A7R (5′-SASGTCVCCSGTSCGGTAS-3′), targeting NRPS A domain sequences. The DNA amplification was done using the thermocycler following the PCR protocol conditions outlined in Table 2. The PCR products were visualised on gel electrophoresis.

**Table 2:** PCR conditions for amplification of the PKS-I, PKS-II and NRPS genes

Primers	Initial denaturation	Cycles	Initial denaturation	Annealing	Extension	Final extension	Hold
KSF/KSR	94 °C, 4 min	32	94 °C, 1 min	56.9 °C, 30 s	72 °C, 1 min and 30 s	72 °C, 10 min	4 °C
KSAF/KSAR	94 °C, 4 min	32	94 °C, 1 min	61.1 °C, 30 s	72 °C, 1 min and 30 s	72 °C, 10 min	4 °C
A3F/A7R	94 °C, 4 min	35	94 °C, 1 min	56.9 °C, 30 s	72 °C, 1 min and 30 s	72 °C, 10 min	4 °C

#### Bioflocculant extraction

The bioflocculant extraction and purification was done as described by Tsilo et al. (2022). The isolate was cultured in 1 L of optimised medium, and the broth was centrifuged (8000 rpm, 4 °C, 30 min) to obtain a cell-free supernatant. Equal volume of sterile water was added, re-centrifuged, then two volumes of ice-cold ethanol were added (2:1, v/v) and precipitated at 4 °C for 12 h. The crude bioflocculant was vacuum dried, re-dissolved in 100 mL sterile water, then mixed with butanol:chloroform (5:2, v/v). After standing 12 h at room temperature, the supernatant was discarded, and the purified precipitate was vacuum dried. Yield was expressed in g/L.

### Bioflocculant characterisation

#### Total carbohydrate and protein contents

The total sugar content was determined by the phenol-sulfuric acid method using glucose as a standard (DuBois et al. 1956), with absorbance measured at 490 nm. Protein content was assessed using the BCA kit (Sigma-Aldrich, South Africa) following the manufacturer’s instructions, with bovine serum albumin (BSA) as the standard and absorbance read at 562 nm using a Jenway 7205 UV/Vis spectrophotometer.

#### Surface morphology and elemental composition of the bioflocculant

A scanning electron microscope (SEM-Sipma-VP03-67, Zeiss) equipped with an elemental analyser was used to examine the surface morphology and elemental composition of the bioflocculants. Samples were rinsed with phosphate buffer saline (0.01 M, pH 7.4), fixed overnight in 3% glutaraldehyde, followed by osmium tetroxide. After dehydration in graded ethanol (20–100%), samples were CO₂–dried. Approximately 0.5 mg of each was mounted on a silicon-coated slide using a spin coater (1000 rpm, 1 min) before SEM imaging and elemental analysis (Li et al. 2022; Khadhraoui et al. 2019).

#### Analysis of functional groups of the bioflocculant

The functional groups of the bioflocculant were identified using Fourier transform infrared (FTIR) spectroscopy (Perkin Elmer System 2000, Cambridge, England) to evaluate the active groups responsible for bioflocculation. The pure bioflocculant was ground to fine powder and placed on the sample holder and the sample spectrum collected. The spectra were recorded with a resolution of 4 cm^–1^ which was conducted over a wavenumber range of 4000 to 350 cm^−1^ (Hua et al. 2021). The functional groups were then assigned to the absorption bands using a spectral library by Bruker optics.

#### Crystallinity of the bioflocculant

The crystallinity of the bioflocculants was analysed using powder X-ray diffraction (XRD). The patterns were recorded with a Bruker D8 Advance diffractometer, which was equipped with Cu–Kα radiation (*λ* = 1.5406 Å) and operated at 40 kV and 40 mA at room temperature. The bioflocculant sample was ground and placed in an aluminium holder and placed into the machine sample assembly and scanned between 5 to 90° with a scan rate of 0.03°/s and a step size of 0.00657°. The Bruker diffraction Eva program was used to evaluate and process the XRD scan (Muthulakshmi et al. 2023).

#### Pyrolysis property of the bioflocculant

The thermogravimetric analysis (TGA) of the bioflocculants was carried out with TGA instrument (Model: DTG-60; Shimadzu, Japan) to determine the degradation behaviour of the bioflocculant during pyrolysis (Okaiyeto et al. 2016). The study was performed in an inert atmosphere (nitrogen) from 25 to 800 °C. The heating rate was uniform at 5 °C/min. The dry, powdered bioflocculant samples were placed in the TGA, and the weight loss as a function of temperature was recorded. Thereafter, the pyrolysis parameters (onset temperature, peak temperature and residual weight) were calculated.

### Application of the bioflocculants in wastewater treatment

The bioflocculant was used to treat brewery and sewage wastewater using the previously optimised operational conditions pH 10, (CaCl_2_ (2%), settling time (10 min) and bioflocculant dosage size (0.5 mg/mL)) (unpublished). The pH of both wastewaters was adjusted to pH 10 using 0.1 M NaOH and 0.1 M HCl. The aluminium sulphate and polyaluminium chloride were utilised as positive controls. The removal efficiencies of the flocculants on chemical oxygen demand (COD) and turbidity were measured by Spectroquant COD test kit and turbidimeter Hanna HI801-01. The removal efficiency (RE) was arithmetically calculated and expressed as percentage RE (%RE) using Eq. 2:

2 $$\%RE=\left(W_o-W\right)/W_o\times100$$

whereby *W*_o_ and *W* represented the parameter values obtained before and after treatment, respectively (Dlamini et al. 2019).

### Data analysis

All experiments were conducted in triplicate, and the results were presented as mean values with their corresponding standard deviations. Statistical analysis was performed using one-way analysis of variance (ANOVA) with GraphPad Prism™ 9, considering a *p* value less than 0.05 as the threshold for statistical significance. Tukey’s honest significant difference test was applied to compare group means for significant differences.

## Results

### Isolation of bioflocculant producer

Eighteen bacterial colonies were isolated from *A. digitata* roots. Amongst the 18 isolates, Gt18 had the highest flocculating activity of 99%. Therefore, the isolate Gt18 was selected and utilised for further experiments due to its consistent and robust flocculation performance across repeated tests.

#### Biochemical characteristics of Isolate Gt18

**Fig. 2:**
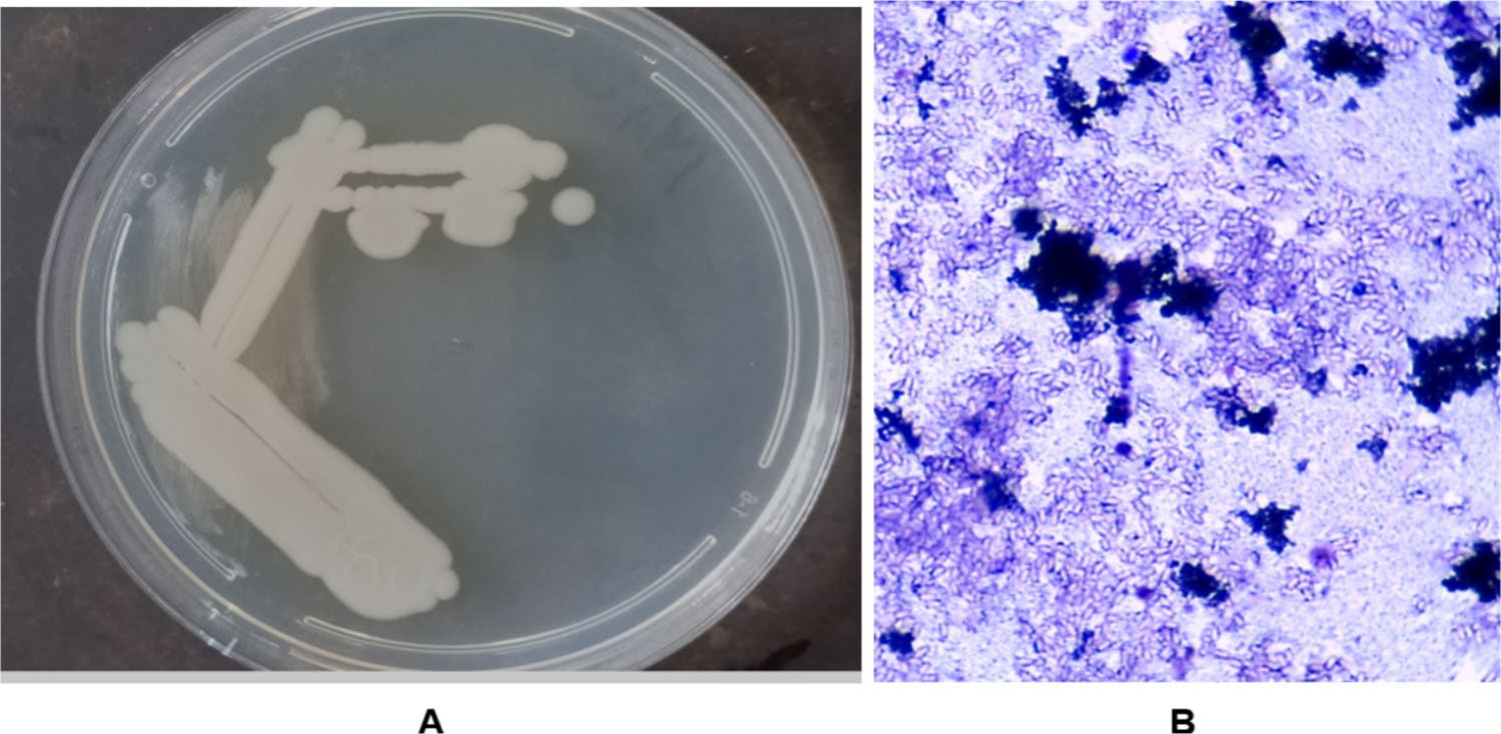
The morphological (**A**) and Gram stain reaction (**B**) of the selected highest bioflocculant-producing isolate

The selected bacterial isolate had a smooth, round, and cream-white appearance as shown in Fig. 2A. The bacterial strain was rod-shaped and Gram-positive, as shown in Fig. 2B.

Biochemical tests were further conducted on the selected bacterial strain, and the results are presented in Table 3. The fermentation studies displayed that the chosen bacterial strain was able to ferment glucose, lactose, and maltose as carbon sources.

**Table 3:** Biochemical characterisation of the bioflocculant-producing endophytic bacterium isolated from *A. digitata* roots

Biochemical test	Isolate Gt18
Spore test	+
Citrate	+
Indole	_
Oxidase	_
Voges-Proskauer	+
Nitrate reduction	+
Catalase	+
Glucose	+
Lactose	+
Mannitol	_
Raffinose	_
Maltose	+
Xylose	_

Key: + : positive, -: negative

#### Molecular identification of of isolate Gt18

The 16S rRNA gene sequencing was used to confirm the identity of the selected isolate. Based on the BLAST results, the selected bacterial isolate was shown to have 99% similarity to *Bacillus albus* with accession number PX048744*.*

#### Cluster genes responsible for bioflocculant production

**Fig. 3:**
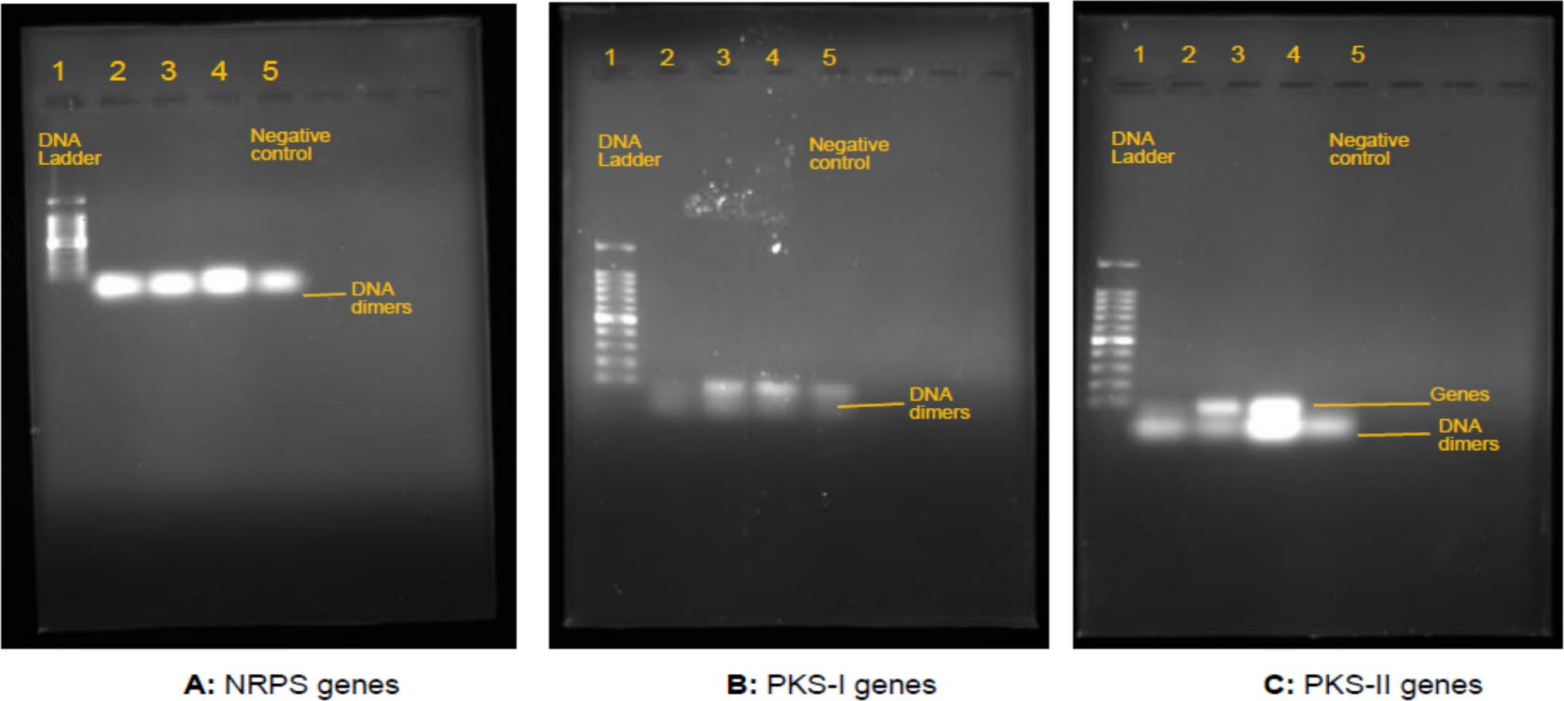
Gel electrophoresis images of PCR gene products of *B. albus* run in triplicate for each primer pair. Lane 1: 1000 bp DNA ladder; Lane 2–4, PCR products of **A** NRPS gene cluster, **B** PKS-I gene cluster, and **C** PKS-II gene cluster. Lane 5: negative control (without template DNA)

The presence of the bioflocculant-producing genes was detected using appropriate primers targeting PKS-I KS, methyl malonyl transferase domains, PKS-II KSa genes and NRPS A domain sequences, and the results are shown in Fig. 3. The selected bacterial strain was found to be positive for one biosynthetic gene cluster PKS-II. The presence of this amplified gene was indicated by the two bands observed in lane 2 and 4, as displayed in Fig. 3C. The other two gene clusters were not detected, and only gene dimers were observed (Fig. 3A, B).

#### Extraction of the bioflocculant

**Fig. 4:**
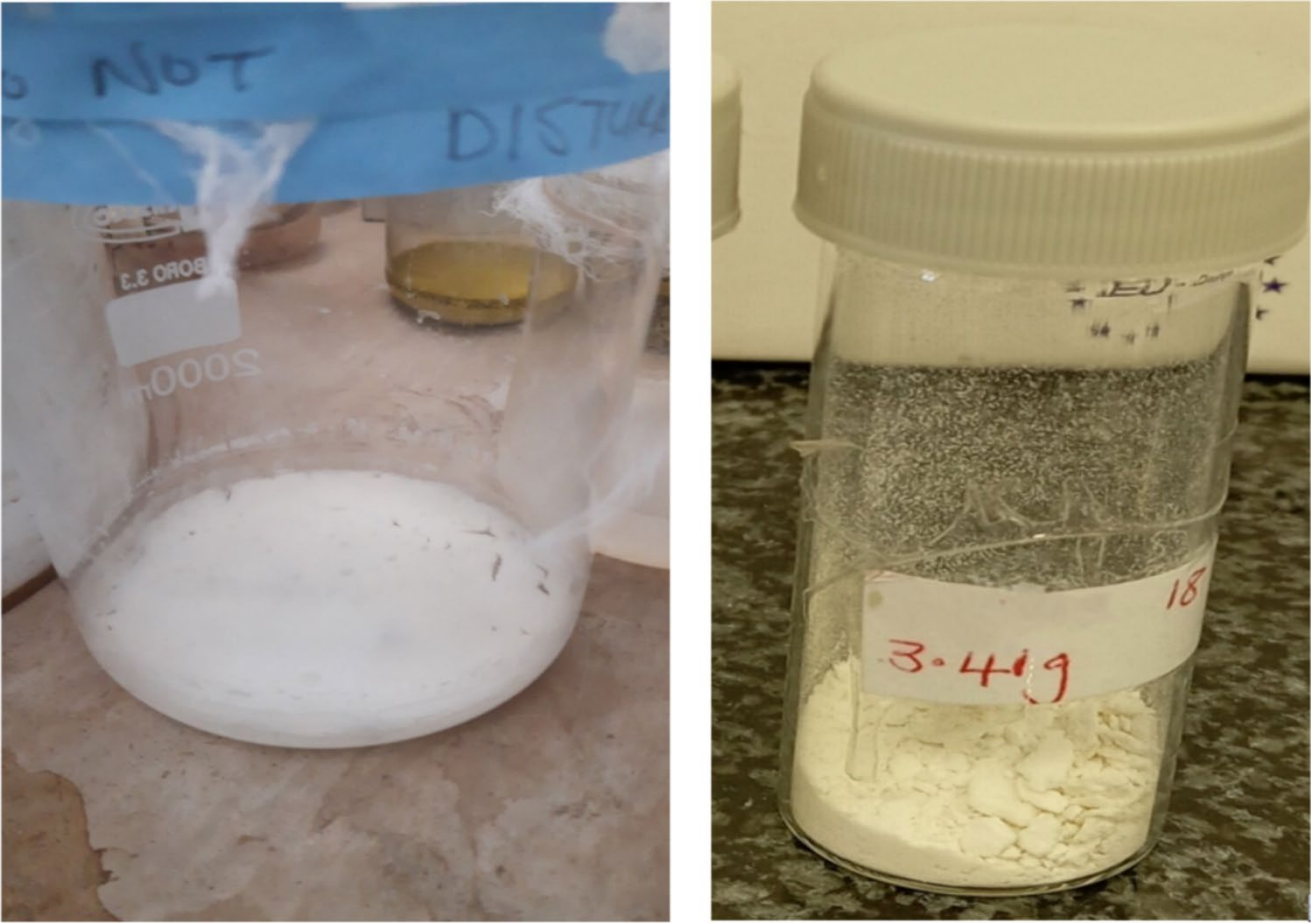
The crude (**A**) and purified (**B**) bioflocculant

The bioflocculant was extracted from *B. albus* using ethanol extraction. The extraction process of the crude and purified bioflocculants is illustrated in Fig. 4. Figure 4A, B shows the dried crude and purified bioflocculant, respectively. The yield of the purified bioflocculant was 3.41 g/L.

#### Chemical composition of the bioflocculants

The chemical composition of the purified bioflocculant was assessed. The chemical analysis revealed that the bioflocculant of *B. albus* contained 80% sugar and 6% proteins.

#### Functional groups of the bioflocculants

**Fig. 5:**
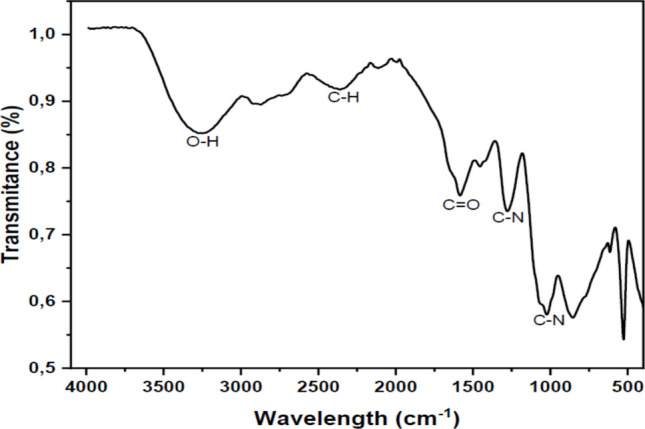
FTIR spectrum of bioflocculant derived from *B. albus*

The FTIR spectra of the bioflocculants produced by *B. albus* are displayed in Fig. 5. The broad stretching absorption peak at around 3400 cm^−1^ indicates the presence of hydroxyl and amino groups. The small peak observed around 2300 cm^−1^ suggested the presence of aliphatic C-H stretch. A distinctive peak at 1650 cm^−1^ corresponds to the carbonyl group, which may arise from carboxylic acids or amide linkages. Additionally, the weak absorption peaks observed around 1200 cm^−1^ confirmed the presence of aromatic and aliphatic amide groups, respectively. The peak at 1020 cm^−1^ indicated the C–N stretch.

#### Morphological analysis of the bioflocculant

**Fig. 6:**
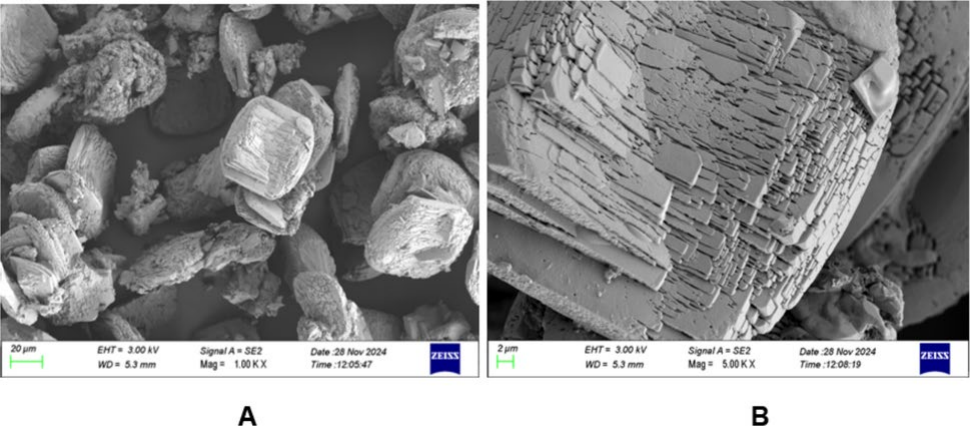
SEM images for the purified bioflocculant of *B. albus*

The surface morphology structure of the purified bioflocculant is presented in Fig. 6. The SEM images indicated that the purified bioflocculant of *B. albus* consisted of square-like shapes (Fig. 6A) made of plate-like rods of regular pattern (Fig. 6B).

#### Elemental composition of the bioflocculant

**Fig. 7:**
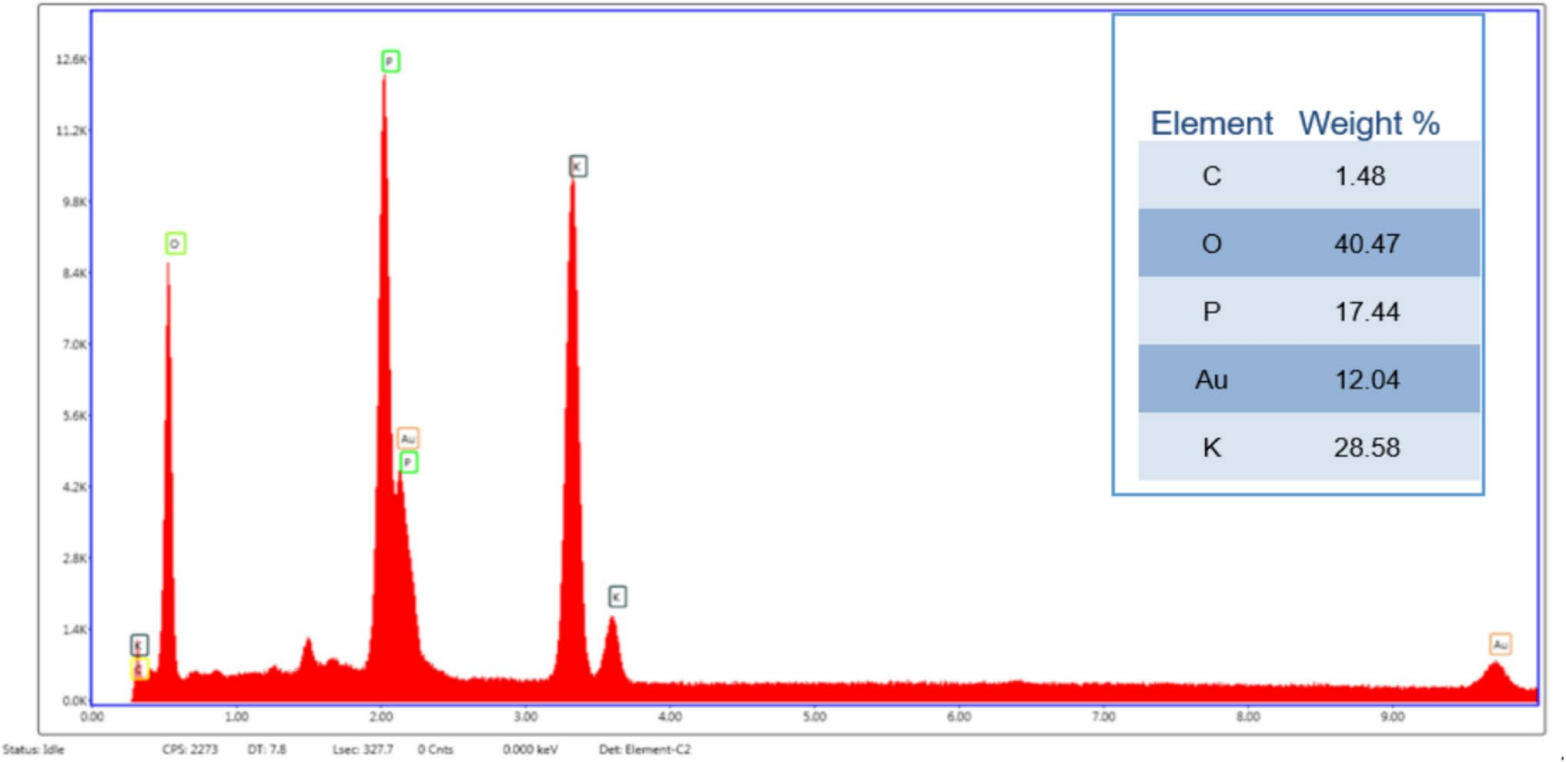
Elemental analysis for bioflocculant of *B. albus*

The EDX analysis of the bioflocculant of *B. albus* is presented in Fig. 7. The EDX analysis revealed the elemental composition in mass proportion (% w/w). The peaks indicate the presence of elements and their intensities (height) relate to their abundance. The elemental spectrum shows the absorption peaks at a mass fraction of C:O:P:K detected in 1.48:40.47:17.44:28.58 (% w/w), respectively.

#### Crystallinity of the bioflocculants

**Fig. 8:**
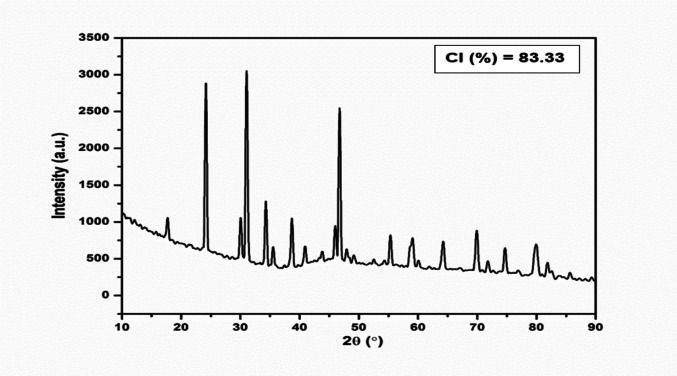
X-ray diffraction patterns of the purified bioflocculant of *B. albus*

The XRD analysis of the purified bioflocculant is presented in Fig. 8. The three prominent diffraction peaks at 2*θ* = 24°, 31°, and 47°, corresponding to (111), (200), and (220) crystal planes, respectively, suggested the presence of a crystalline lattice within the bioflocculant. Overall, the crystallinity index (CI) was estimated to be about 83.33%, signifying a crystalline nature bioflocculant.

#### Pyrolysis profile of the bioflocculant

**Fig. 9:**
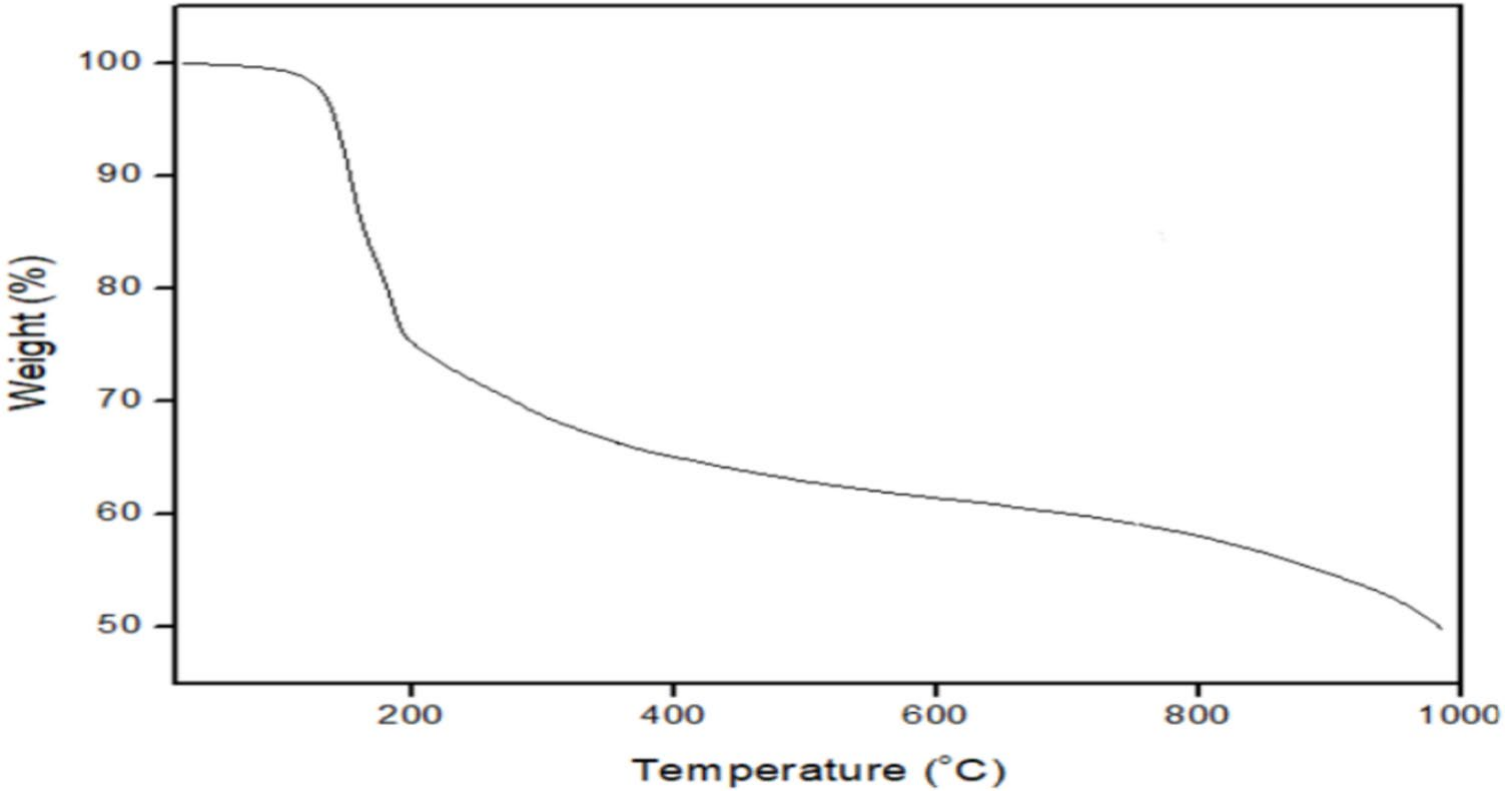
The TGA spectrum of the purified bioflocculant of *B. albus*

The pyrolysis profile of the bioflocculant is shown in Fig. 9. The thermograph displays different stages of weight loss. The bioflocculant exhibited an initial weight loss of 2% at temperatures between 30 and 180 °C. The first significant weight loss of 24% occurred at a temperature between 180 and 250 °C. The second gradual decomposition phase was observed at 250–600 °C, accounting for 14% mass loss. The final steady decline in weight loss was observed between 600 and 998 °C, resulting in about 50% final residue.

#### Efficiency of bioflocculant in wastewater treatment

The removal efficiencies of the flocculants on COD and turbidity were assessed, and the results are displayed in Table 4. The bioflocculant performed significantly better in COD removal (*p* value < 0.05) in comparison to aluminium sulphate and polyaluminium chloride in both wastewaters. It had the highest COD removal efficiencies of 70 and 57% on sewage and brewery wastewater, respectively. Moreover, it performed relatively the same (*p* value > 0.05) with the other tested flocculants on turbidity reduction of sewage water, giving 99.8% activity. However, it surpassed aluminium sulphate on turbidity reduction on brewery wastewater and illustrated the efficiency of 75%.

**Table 4:** The removal efficiency of the bioflocculant from *B. albus* in comparison to chemical flocculants

Flocculant	RE ± SD (%)			
	Sewage wastewater		Brewery wastewater	
	COD	Turbidity	COD	Turbidity
Bioflocculant	70 ± 0.21^a^	99.8 ± 0.09^a^	57 ± 0.38^a^	75 ± 0.08^a^
Aluminium sulphate	36 ± 0.06 ^b^	97.8 ± 0.11^a^	38 ± 0.07^b^	47 ± 0.13^b^
Polyaluminium chloride	47 ± 0.33^b^	99.1 ± 0.05^a^	38 ± 0.41^b^	72 ± 0.04^a^

The superscripts signify statistical significance at *p* < 0.05 where values sharing the same letter are not significantly different (*p* > 0.05)

## Discussion

In this study, out of 18 bacterial endophytic isolates, Isolate Gt18 illustrated the highest bioflocculant production, confirming *A. digitata* as a reservoir of potent bioflocculant producers. This aligned with the study by Saha et al. (2024), who reported endophytic microbial bioflocculant from *Globba marantina,* further underscoring plants as potent sources of endophytic bioflocculant producers*.*

Isolate Gt18 was identified as the member of the *Bacillus* species, namely *Bacillus albus*. Thus, *B. albus* belongs to the phylum Firmicutes, which primarily consists of Gram-positive, rod-shaped, spore-forming and facultatively anaerobic microorganisms (Abu Tawila et al. 2018). The genus *Bacillus* consists of a diversity of bacterial species that have shown high capabilities of secreting effective bioflocculants (El-Rouby et al. 2015). For instance, *Bacillus clausii* NB2 (Bukola and Gboyega 2014), *Bacillus subtilis* ZHX3 (Xia et al. 2022) and *Bacillus pumilus* (Maliehe et al. 2020), *Bacillus cereus* S55 (Harinisri et al. 2024*)*, *Bacillus licheniformis* (Ramadhani et al. 2022*)* and *Bacillus megaterium* (Pu et al. 2020) have been reported to be potent bioflocculant producers. Nevertheless, according to our knowledge, *B. albus* has not been reported to produce bioflocculant*.*

The biosynthesis of microbial flocculants is a complex process that involves multistage with many biosynthetic genes and enzymes such as NRPSs and PKSs. Therefore, in this study, the gene clusters responsible for the synthesis of bioflocculants were investigated to understand the mechanisms behind bioflocculant production. *B. albus* was found to be positive for the gene cluster PKS-II, clustered on a 10 kb DNA segment. The PKS-II gene cluster mediates the biosynthesis of polyketide secondary metabolites such as bioflocculants by combinatorial functioning of various domains (Selvin et al. 2016; McBride et al. 2023). Therefore, its detection in this study implied that this cluster of genes might have been expressed during bioflocculant production by *B. albus*. However, further confirmatory studies are needed to support this claim. To our surprise, PKS-I and NRPS gene clusters were not detected, implying that they were not responsible for bioflocculant production by *B. albus*.

Bioflocculant productivity and yields are good qualitative indicators of how bacterial cells effectively convert culture medium components into bioflocculants (Maliehe 2018). In this study, *B. albus* yielded 3.41 g/L after 72 h of fermentation. This was considered a good yield as most bioflocculants produced by bacterial strains are often less than 2 g/L. For instance, the *B. albus’* bioflocculant yield was significantly higher than the 1.15 g/L obtained from *Bacillus firmus* (Salehizadeh and Shojaosadati 2002), 1.36 g/L yield derived from *Proteus mirabilis* (Arafa et al. 2014) and 2.0 g/L derived from *Bacillus velezensis* (Hassimi et al. 2020). The high yield might be attributed to *B. albus’* ability to optimally survive in the set culture conditions and the expressed flocculation-related gene cluster as well as the polarity of the solvents that were used during extraction and purification processes.

The chemical composition revealed the bioflocculant to have a high content of total carbohydrates and low total protein content, suggesting it to be a glycoprotein biopolymer (Cherian et al. 2025). The carbohydrates are primarily composed of polysaccharides, which play a significant role in the flocculating activity of the bioflocculant due to their sticky nature and bridging mechanism which enhances effective particle aggregation (Wang et al. 2024). Furthermore, the high carbohydrate content suggested that the bioflocculant is a polysaccharide-rich biopolymer and is presumed to be the most responsible component for the high flocculating activity. Furthermore, the high carbohydrate content might attribute to the high thermal stability of this bioflocculant (Agunbiade et al. 2017). The proteins likely contribute to the structural stability and the interactions of the functional groups during the flocculation process. The other 14% may consist of other constituents such as uronic acids, nucleic acids, lipids and inorganic ions, which may assist in charge neutralisation, metal ion binding and enhancing flocculation efficiency (Mohammed and Dagang 2019). The findings aligned with the studies by Ntsangani et al. (2017) and Agunbiade et al. (2022), who both reported the bioflocculants obtained from *Bacillus sp.* AEMREG4 and *Bacillus velezensis* to mainly compose of carbohydrates.

The various functional groups found on molecular chains of bioflocculants are responsible for the flocculation performance, especially in binding and aggregation of the suspended particles (Kurniawan et al. 2022). The observed functional groups (hydroxyl (-OH), carbonyl (C = O) and amino groups (C-N)) were presumed to have contributed to the flocculating activity and enhanced the stability of the bioflocculant from *B. albus*. The presence of carbonyl and C-N stretch indicated the occurrence of polysaccharide backbone (glycosidic linkage). Moreover, the amide and amino groups affirmed the presence of proteinaceous nature of the bioflocculant. Thus, the observed functional groups suggested the bioflocculant to be a glycoprotein (composing of polysaccharides and proteins) molecule (Maliehe 2018). The findings were similar to those of Wu et al. (2023) and Joshi et al. (2019), who found hydroxyl and amino groups from bioflocculants of *Bacillus thuringiensis* and *Bacillus licheniformis* NJ3, further confirming these groups as crucial in the bioflocculation process. Dai et al. (2024) also reported the presence of carbonyl groups which enhanced the adsorption properties of *Bacillus subtilis’* bioflocculant in dye removal.

The surface morphology plays a significant role in the effectiveness or poor flocculating activities of the bioflocculant (Li et al. 2021). The plate-like shape of the bioflocculant from *B. albus* provided a broad surface area for the adsorption of suspended particles. These characteristics influenced its ability to bridge particles, stabilise flocs and ultimately improve the efficiency of the flocculation process (Srivastava et al. 2018). A similar morphology was observed in a bioflocculant produced by *Bacillus velezensis* (Binmad et al. 2022).

The elemental composition of the bioflocculants determines their flexibility or stability (Selepe and Maliehe 2024). Carbon was detected at a low energy range; however, its presence confirmed the organic nature of the bioflocculant. Carbon is a primary element in polysaccharides and proteins, and it is responsible for forming the backbone of the biopolymeric structure (Su et al. 2021). Its presence implied that the bioflocculant consisted of carbon-rich macromolecules such as exopolysaccharides (Atmakuri et al. 2024). For instance, Botirova et al. (2023) reported *Bacillus simplex* from soil as an exopolysaccharide-based bioflocculant, which played a role in the bioflocculant’s stability and flocculation efficiency. The high presence of oxygen suggested a high content of oxygenated functional groups, which in this study included the hydroxyl and carbonyl groups, which are preferred for flocculation (Vimala et al. 2020). The finding corroborated the report of Giri et al. (2015), in which the elemental analysis of the bioflocculant of *Bacillus subtilis* revealed oxygen in a high weight fraction of 54.7%. The detection of phosphorus implied the presence of phosphate groups, which may originate from nucleic acids within the bioflocculant (Kaur et al. 2019). The potassium peaks observed may be due to the residual ions from the culture medium used during bioflocculant production and may not play any significant role in the structure of the bioflocculant (Dlamini et al. 2020). Gold was also detected; however, it is not naturally present in biological materials. Its presence may be attributed to the gold coating process used during SEM/EDX sample preparation to enhance conductivity; therefore, it is not considered part of the bioflocculant composition.

The XRD analysis of the *B. albus’* bioflocculant provided an insight into the degree of crystallinity, molecular arrangement and possible presence of inorganic residues. The XRD pattern showed prominent sharp peaks, indicating that the bioflocculant exhibits a crystalline nature. The sharp peaks may be due to the highly ordered polysaccharide chains, mineral residues and proteinaceous crystallites, possibly due to hydrogen bonding amongst functional groups (Song et al. 2021), which play a crucial role in improving mechanical strength and stability (Okaiyeto et al. 2015). This correlated with a study by Harinisri et al. (2024), where *Bacillus cereus* (S55) produced a crystalline nature bioflocculant.

The different thermal profiles of the bioflocculant were assessed to ascertain its thermal stability. The initial 2% weight loss observed was due to loss of moisture and loosely bound volatile components such as the hydroxyl group (Okaiyeto et al. 2016). Similarly, Mohite et al. (2019) and Artifon et al. (2023) reported the weight loss due to loss of moisture from bioflocculants. Additionally, the weight loss at less than 180 °C indicated that the bioflocculant can withstand moderate heat before starting to degrade. The first major decomposition of 24% was likely attributed to the thermal degradation of low molecular weight polysaccharides or proteins. This phase represented the decomposition of the bioflocculant’s less stable organic fractions. The second decomposition phase was attributed to the breakdown of more thermally stable functional groups such as the carboxylic acid, reflecting the degradation of the primary polysaccharide or glycoprotein backbone. After 998 °C, about 50% of the bioflocculant was obtained, confirming the moderate thermal stability of the bioflocculant, which was greatly attributed to the high carbohydrate composition (Sun et al. 2012). However, no true plateau or complete stabilisation was observed.

The removal efficiency of bioflocculant in comparison to used conventional chemical flocculants (aluminium sulphate and polyaluminium chloride) was assessed. The comparatively higher COD and turbidity removal efficiencies demonstrated by the bioflocculant in comparison to the tested conventional flocculants implied that the bioflocculant from *B. albus* has potential to be used interchangeably with the conventional flocculants in wastewater treatment. This is the good news as this can mitigate the adverse effect implicated with the predominant utilisation of the chemical flocculant. The findings agreed with those reported by Mburu et al. (2024) and Agunbiade et al. (2022), where bioflocculants performed significantly better than conventional flocculants in reduction of COD and turbidity in wastewater. However, the lower efficiencies shown by conventional flocculants used in this study might be due to the fact that their operational conditions were not previously optimised but only for the bioflocculant.

## Conclusion

This study confirmed that endophytic bacteria are excellent bioflocculant producers. Out of 18 selected isolates, Isolate Gt18, which was identified as *B. albus*, demonstrated the flocculating activity of 99% against kaolin clay solution. The bioflocculant production was attributed to the presence of a type II PKS biosynthetic gene cluster. *B. albus* yielded promising bioflocculant of 3.41 g/L, which was a glycoprotein with predominant carbohydrate composition. It had a plate-like structure and was composed of hydroxyl, amine, and carbonyl groups as the main functional groups. The bioflocculant was found to have a moderate heat-stable and semicrystalline profile. The bioflocculant illustrated high reduction efficiencies on COD and turbidity of the tested sewage and brewery wastewater. For future studies, the mechanism of action of the bioflocculant from *B. albus* in the wastewater treatment process ought to be assessed.

## Data Availability

The bacterium identified in this study is available from GenBank [https://submit.ncbi.nlm.nih.gov/subs/?search=SUB15505987]. Its accession number is provided in this manuscript. The other datasets used during this study are available from the corresponding author on request.

## References

[CR1] Abu Tawila ZM, Ismail S, Dadrasnia A, Usman MM (2018) Production and characterization of a bioflocculant produced by *Bacillus salmalaya* 139SI-7 and its applications in wastewater treatment. Molecules 23(10):2689 30340415 10.3390/molecules23102689 PMC6222882

[CR2] Agunbiade MO, Van Heerden E, Pohl CH, Ashafa AT (2017) Flocculating performance of a bioflocculant produced by *Arthrobacter humicola* in sewage waste water treatment. BMC Biotechnol 17(1):51 28606076 10.1186/s12896-017-0375-0 PMC5469021

[CR3] Agunbiade M, Oladipo B, Ademakinwa AN, Awolusi O, Adesiyan IM, Oyekola O, Ololade O, Ojo A (2022) Bioflocculant produced by *Bacillus velezensis* and its potential application in brewery wastewater treatment. Sci Rep 12(1):10945 35768624 10.1038/s41598-022-15193-8 PMC9243052

[CR4] Alias J, Hasan HA, Abdullah SRS, Othman AR (2022) Properties of bioflocculant-producing bacteria for high flocculating activity efficiency. Environ Technol Innov 27:102529

[CR5] Arafa RA, El-Rouby MN, Abass HA, El-Khier Z (2014) Bioflocculants produced by bacterial isolates from Egyptian soil 1-characterization and application of extracellular bioflocculants and nanoparticles for treatment of river Nile water. J Pharm Biol Sci 9(5):103–114

[CR6] Artifon W, Immich APS, da Silva A, de Souza AAU, de Oliveira D (2023) A novel bioflocculant extracted from excess-activated sludge for dye-containing wastewater treatment. J Water Process Eng 56:104568

[CR7] Atmakuri A, Yadav B, Tiwari B, Drogui P, Tyagi RD, Wong JW (2024) Nature’s architects: a comprehensive review of extracellular polymeric substances and their diverse applications. Waste Dispos Sust En 6(4):529–551

[CR8] Binmad S, Numnuam A, Kaewtatip K, Kantachote D, Tantirungkij M (2022) Characterization of novel extracellular polymeric substances produced by *Bacillus velezensis* P1 for potential biotechnological applications. Polym Adv Technol 33(8):2470–2479

[CR9] Botirova G, Amriddinova D, Yili NRA (2023) Isolation and identification of exopolysaccharide-based bioflocculant of soil bacteria *Bacillus simplex* PBB-17. ILMIY Axborotnoma 135(118):5–8

[CR10] Bukola AT, Gboyega AE (2014) Production and characterization of bioflocculant produced by Bacillus clausii NB2. Innov Rom Food Biotechnol 14:13–25

[CR11] Chen Z, Liu P, Li Z, Yu W, Wang Z, Yao H, Wang Y, Li Q, Deng X, He N (2017) Identification of key genes involved in polysaccharide bioflocculant synthesis in Bacillus licheniformis. Biotechnol Bioeng 114(3):645–655 27667128 10.1002/bit.26189

[CR12] Cherian T, Eranhottu S, Mohanraju R (2025) Process optimization and bioflocculative insights of glycoprotein bioflocculant produced by marine bacterium *Bacillus oceanisediminis* LBB1. Biocatal Agric Biotechnol 65:103555

[CR13] Chigede N, Chikwambi Z, Mpofu ID, Madzimure J (2024) Isolation and characterization of biosurfactant-producing microbes isolated from the gastrointestinal system of broiler birds fed a commercial diet. Anim Biotechnol 35(1):2263771 37814822 10.1080/10495398.2023.2263771 PMC12674367

[CR14] Costa LEDO, Queiroz MVD, Borges AC, Moraes CAD, Araújo EFD (2012) Isolation and characterization of endophytic bacteria isolated from the leaves of the common bean (*Phaseolus vulgaris*). Braz J Microbiol 43:1562–1575 24031988 10.1590/S1517-838220120004000041 PMC3769033

[CR15] Dai J, Zhao X, Mu S, Yang Q, Zhao C, Zhao Z (2024) A novel polysaccharide-based bioflocculant produced by Bacillus subtilis 35A and its application in the treatment of dye decolorization, heavy metal ion adsorption and meat product wastewater. Front Microbiol 15:1457909 39238890 10.3389/fmicb.2024.1457909 PMC11374711

[CR16] Dlamini NG, Basson AK, Pullabhotla VSR (2019) Optimization and application of bioflocculant passivated copper nanoparticles in the wastewater treatment. Int J Environ Res Public Health 16:2185 31226768 10.3390/ijerph16122185 PMC6616601

[CR17] Dlamini NG, Basson AK, Pullabhotla RV (2020) Wastewater treatment by a polymeric bioflocculant and iron nanoparticles synthesized from a bioflocculant. Polymers 12(7):1618 32708211 10.3390/polym12071618 PMC7407570

[CR18] DuBois M, Gilles KA, Hamilton JK, Rebers PA, Smith F (1956) Colorimetric method for determination of sugars and related substances. Anal Chem 28(3):350–356

[CR19] El-Rouby MN, Abass HA, Fattah SMA, Arafa RA, Khattab AENA, El-Khier ZAA (2015) Bioflocculants produced by isolated bacteria from Egyptian soil II-cytopathological effect of extracellular and intracellular bacterial extracts. Glob J Biotechnol Biochem 10(2):47–61

[CR20] Etminani F, Harighi B (2018) Isolation and identification of endophytic bacteria with plant growth promoting activity and biocontrol potential from wild pistachio trees. Plant Pathol J 34(3):208 29887777 10.5423/PPJ.OA.07.2017.0158 PMC5985647

[CR21] Fu L, Jiang B, Wei J, Liu J, Hu X, Zhang L (2020) Transcriptome analysis of polysaccharide-based microbial flocculant MBFA9 biosynthesis regulated by nitrogen source. Sci Rep 10(1):2918 32075995 10.1038/s41598-020-59114-z PMC7031244

[CR22] Giri SS, Harshiny M, Sen SS, Sukumaran V, Park SC (2015) Production and characterization of a thermostable bioflocculant from *Bacillus subtilis* F9, isolated from wastewater sludge. Ecotoxicol Environ Saf 121:45–50 26091955 10.1016/j.ecoenv.2015.06.010

[CR23] Hakim S, Naqqash T, Nawaz MS, Laraib I, Siddique MJ, Zia R, Mirza MS, Imran A (2021) Rhizosphere engineering with plant growth-promoting microorganisms for agriculture and ecological sustainability. Front Sustain Food Syst 5:617157

[CR24] Hardoim PR, van Overbeek LS, van Elsas JD (2008) Properties of bacterial endophytes and their proposed role in plant growth. Trends Microbiol 16(10):463–471 18789693 10.1016/j.tim.2008.07.008

[CR25] Harinisri K, Prathiviraj R, Selvi BT (2024) Screening, characterization, and production of *Bacillus cereus* (S55) bioflocculant isolated from soil for application in wastewater treatment. Biotechnol Lett 5(2024):151–164 10.1016/j.biotno.2024.11.003 PMC11615589 39633682

[CR26] Hassimi AH, Hafiz RE, Muhamad MH, Abdullah SRS (2020) Bioflocculant production using palm oil mill and sago mill effluent as a fermentation feedstock: characterization and mechanism of flocculation. J Environ Manage 260:110046 32090804 10.1016/j.jenvman.2019.110046

[CR27] Hua JQ, Zhang R, Chen RP, Liu GX, Yin K, Yu L (2021) Energy-saving preparation of a bioflocculant under high-salt condition by using strain *Bacillus* sp. and the interaction mechanism towards heavy metals. Chemosphere 267:129324 33352365 10.1016/j.chemosphere.2020.129324

[CR28] Joshi N, Rathod M, Vyas D, Kumar R, Mody K (2019) Multiple pollutants removal from industrial wastewaters using a novel bioflocculant produced by *Bacillus licheniformis* NJ3. Environ Prog Sustain Energy 38(s1):S306–S314

[CR29] Kaur R, Roy D, Yellapu SK, Tyagi RD, Drogui P, Surampalli RY (2019) Enhanced composting leachate treatment using extracellular polymeric substances as bioflocculant. J Environ Eng 145(11):04019075.Ka

[CR30] Khadhraoui M, Sellami M, Zarai Z, Saleh K, Rebah FB, Leduc R (2019) Cactus juice preparations as bioflocculant: properties, characteristics and application. Environ Eng Manag J 18(1):137–146

[CR31] Khumalo S, Kudanga T, Nyanhongo G, Ngema S, Maliehe T, Madoroba E (2025) Marine *Streptomyces* reveals anti-*Staphylococcus* activity through interfering with respiratory chain dehydrogenase activity and membrane integrity. Discov Appl Sci 7:171

[CR32] Kumar S, Yadav V, Nehra A (2023) A review of characterization tools and techniques for nanomaterials and nanocomposites. Int J Psychosoc Rehabil 27(2):42–53

[CR33] Kurniawan SB, Imron MF, Chik CENCE, Owodunni AA, Ahmad A, Alnawajha MM, Rahim NFM, Said NSM, Abdullah SRS, Kasan NA, Ismail S (2022) What compound inside biocoagulants/bioflocculants is contributing the most to the coagulation and flocculation processes? Sci Total Environ 806:150902 34653447 10.1016/j.scitotenv.2021.150902

[CR34] Li NJ, Lan Q, Wu JH, Liu J, Zhang XH, Zhang F, Yu HQ (2021) Soluble microbial products from the white-rot fungus *Phanerochaete chrysosporium* as the bioflocculant for municipal wastewater treatment. Sci Total Environ 780:146662 34030296 10.1016/j.scitotenv.2021.146662

[CR35] Li L, He Z, Song Z, Sheng T, Dong Z, Zhang F, Ma F (2022) A novel strategy for rapid formation of biofilm: polylactic acid mixed with biofloccula;ltrnt modified carriers. J Clean Prod 374:134023

[CR36] Lodewyckx C, Vangronsveld J, Porteous F, Moore ER, Taghavi S, Mezgeay M, der Lelie DV (2002) Endophytic bacteria and their potential applications. Crit Rev Plant Sci 21(6):583–606

[CR37] Maliehe, TS (2018) Production, Characterisation and application of bioflocculants from pure bacterial strains and their consortia isolated from Sodwana Bay in Kwa-Zulu Natal, South Africa (Doctoral dissertation, University of Zululand)

[CR38] Maliehe T, Basson A, Singh M (2020) Wastewater treatment by a novel bioflocculant from a consortium of *Bacillus pumilus* JX860616 and *Bacillus subtilis* CSM5. Biosci Res 17:1610–1625

[CR39] Manganyi MC, Dikobe TB, Maseme MR (2024) Exploring the potential of endophytic microorganisms and nanoparticles for enhanced water remediation. Molecules 29(12):2858 38930923 10.3390/molecules29122858 PMC11206248

[CR40] Mburu A, Njuguna D, Nzila C, Musieba F, Waithaka A, Mwangi G, Kemunto A (2024) Comparative treatment of textile dye wastewater with chemical coagulants and bacterial exopolysaccharides. J Text Inst 116(9):2100–2110

[CR41] McBride CM, Miller EL, Charkoudian LK (2023) An updated catalogue of diverse type II polyketide synthase biosynthetic gene clusters captured from large-scale nucleotide databases. Microb Genom 9(3):000965 10.1099/mgen.0.000965 PMC10132072 36951894

[CR42] Mohammed JN, Dagang WRZW (2019) Role of cationization in bioflocculant efficiency: a review. Environ Process 6(2):355–376

[CR43] Mohite BV, Koli SH, Rajput JD, Patil VS, Agarwal T, Patil SV (2019) Production and characterization of multifacet exopolysaccharide from an agricultural isolate, *Bacillus subtilis*. Biotechnol Appl Biochem 66(6):1010–1023 31539174 10.1002/bab.1824

[CR44] Muthulakshmi L, Kiran GS, Ramakrishna S, Cheng KY, Ramachandran RA, Mathew MT, Pruncu CI (2023) Towards improving the corrosion resistance using a novel eco-friendly bioflocculant polymer produced from *Bacillus* sp. Mater Today Commun 35:105438

[CR45] Ntsangani N, Okaiyeto K, Uchechukwu NU, Olaniran AO, Mabinya LV, Okoh AI (2017) Bioflocculation potentials of a uronic acid-containing glycoprotein produced by *Bacillus* sp. AEMREG4 isolated from Tyhume River, South Africa. 3 Biotech 7(1):78 10.1007/s13205-017-0695-8 PMC5429313 28500400

[CR46] Nxumalo CI, Ngidi LS, Shandu JSE, Maliehe TS (2020) Isolation of endophytic bacteria from the leaves of *Anredera cordifolia* CIX1 for metabolites and their biological activities. BMC Complement Altern Med 20:300 10.1186/s12906-020-03095-z PMC7541265 33028279

[CR47] Okaiyeto K, Nwodo UU, Mabinya LV, Okoh AI (2015) Characterization and flocculating properties of a biopolymer produced by *Halomonas* sp. Okoh. Water Environ Res 87(4):298–303 26462073 10.2175/106143015X14212658613479

[CR48] Okaiyeto K, Nwodo UU, Mabinya LV, Okoli AS, Okoh AI (2016) Evaluation of flocculating performance of a thermostable bioflocculant produced by marine *Bacillus* sp. Environ Technol 37(14):1829–1842 26797258 10.1080/09593330.2015.1133717

[CR49] Okaiyeto K, Ekundayo TC, Okoh AI (2020) Global research trends on bioflocculant potentials in wastewater remediation from 1990 to 2019 using a bibliometric approach. Lett Appl Microbiol 71(6):567–579 32780872 10.1111/lam.13361

[CR50] Pu L, Zeng YJ, Xu P, Li FZ, Zong MH, Yang JG, Lou WY (2020) Using a novel polysaccharide BM2 produced by *Bacillus megaterium* strain PL8 as an efficient bioflocculant for wastewater treatment. International Int J Biol Macromol 162:374–384 10.1016/j.ijbiomac.2020.06.167 32569694

[CR51] Qi Z, Zhu Y, Guo H, Chen Y, Zhao Y, Zhou Y, Wang X, Yang Y, Qin W, Shao Q (2019) Production of glycoprotein bioflocculant from untreated rice straw by a CAZyme-rich bacterium, *Pseudomonas* sp. HP2. J Biotechnol 306:185–192 31629784 10.1016/j.jbiotec.2019.10.011

[CR52] Rajivgandhi G, Vimala RTV, Maruthupandy M, Alharbi NS, Kadaikunnan S, Khaled JM, Manoharan N, Li WJ (2021) Enlightening the characteristics of bioflocculant of endophytic actinomycetes from marine algae and its biosorption of heavy metal removal. Environ Res 200:111708 34280417 10.1016/j.envres.2021.111708

[CR53] Ramadhani AN, Sarosa ANAKW, Al Rosyad LH (2022) The potency of microbial flocculant produced by *B. licheniformis* using molasses as the carbon source and its application in food industry wastewater treatment. Mater Today Proc 63:S244–S247

[CR54] Roy M, Mohanty K (2020) Valorization of waste eggshell-derived bioflocculant for harvesting *T. obliquus*: process optimization, kinetic studies and recyclability of the spent medium for circular bioeconomy. Bioresour Technol 307:123205 32234589 10.1016/j.biortech.2020.123205

[CR55] Saha S, Santra HK, Chattopadhyay S, Banerjee D (2024) Production of exopolysaccharide from an endophytic *Fusarium* sp. Glos2 and documentation of its in vitro antioxidative potentialities. J Pure Appl Microbiol 18(4):2911–2924

[CR56] Salehizadeh H, Shojaosadati SA (2002) Isolation and characterisation of a bioflocculant produced by *Bacillus firmus*. Biotechnol Lett 24(1):35

[CR57] Salehizadeh H, Yan N, Farnood R, Zheng Y (2018) Recent advances in polysaccharide bio-based flocculants. Biotechnol Adv 36(1):92–119 28993221 10.1016/j.biotechadv.2017.10.002

[CR58] Selepe TN, Maliehe TS (2024) Bioflocculation of pollutants in wastewater using flocculant derived from *Providencia huaxiensis* OR794369. 1. BMC Microbiol 24(1):39 38281910 10.1186/s12866-023-03144-w PMC10823601

[CR59] Selvin J, Sathiyanarayanan G, Lipton AN, Al-Dhabi NA, Valan Arasu M, Kiran GS (2016) Ketide synthase (KS) domain prediction and analysis of iterative type II PKS gene in marine sponge-associated actinobacteria producing biosurfactants and antimicrobial agents. Front Microbiol 7:63 26903957 10.3389/fmicb.2016.00063 PMC4751271

[CR60] Singh R, Pandey KD, Singh M, Singh SK, Hashem A, Al-Arjani ABF, Abd_Allah EF, Singh PK, Kumar A (2022) Isolation and characterization of endophytes bacterial strains of *Momordica charantia* L. and their possible approach in stress management. Microorganisms 10(2):290 10.3390/microorganisms10020290 PMC8877101 35208743

[CR61] Song P, Zhou F, Li F, Han Z, Wang L, Xu J, Zhang B, Wang M, Fan J, Zhang B (2021) Superfine pulverisation pretreatment to enhance crystallinity of cellulose from *Lycium barbarum* L. leaves. Carbohydr Polym 253:117207 33278976 10.1016/j.carbpol.2020.117207

[CR62] Srinivasan KR, Wong JWC, Murugesan K (2023) Production of bioflocculant from *Klebsiella pneumoniae*: evaluation of fish waste extract as substrate and flocculation performance. Envir Technol 44(26):4046–4059 10.1080/09593330.2022.2078672 35567323

[CR63] Srivastava A, Seo SH, Ko SR, Ahn CY, Oh HM (2018) Bioflocculation in natural and engineered systems: current perspectives. Crit Rev Biotechnol 38(8):1176–1194 29631430 10.1080/07388551.2018.1451984

[CR64] Su L, Feng Y, Wei K, Xu X, Liu R, Chen G (2021) Carbohydrate-based macromolecular biomaterials. Chem Rev 121(18):10950–11029 34338501 10.1021/acs.chemrev.0c01338

[CR65] Sun J, Zhang X, Miao X, Zhou J (2012) Preparation and characteristics of bioflocculants from excess biological sludge. Bioresour Technol 126:362–366 23127842 10.1016/j.biortech.2012.08.042

[CR66] Tsilo PH, Basson AK, Ntombela ZG, Maliehe TS, Pullabhotla VR (2022) Production and characterization of a bioflocculant from *Pichia kudriavzevii* MH545928. 1 and its application in wastewater treatment. Int J Environ Res Public Health 19(6):3148 35328836 10.3390/ijerph19063148 PMC8953087

[CR67] Vimala RTV, Escaline JL, Sivaramakrishnan S (2020) Characterization of self-assembled bioflocculant from the microbial consortium and its applications. J Environ Manage 258:110000 31929048 10.1016/j.jenvman.2019.110000

[CR68] Wang L, Zhang X, Zhang X, Hu X, Yang J, Zhang H (2024) Mechanism analysis of a novel natural cationic modified dextran flocculant and its application in the treatment of blue algal blooms. Int J Biol Macromol 254:128002 37949280 10.1016/j.ijbiomac.2023.128002

[CR69] Wu CY, Zhong ZC, Wu XP, Chen YJ, Shu CC (2023) A comprehensive study on flocculation of anionic dyes using the bioflocculant produced by *Bacillus thuringiensis*: kinetics, affecting factors, and perspectives. Water Environ Res 95(5):e10868 37072151 10.1002/wer.10868

[CR70] Xia M, Zhou H, Amanze C, Hu L, Shen L, Yu R, Liu Y, Chen M, Li J, Wu X, Qiu G (2022) A novel polysaccharides-based bioflocculant produced by *Bacillus subtilis* ZHX3 and its application in the treatment of multiple pollutants. Chemosphere 289:133185 34883128 10.1016/j.chemosphere.2021.133185

[CR71] Yang Y, Jiang C, Wang X, Xie Y, Wang D, Xu S, Zhuang X (2025) Advancing bioflocculants for sustainable harmful algal bloom control: mechanisms, applications, and resource valorization. ACS EST Eng 5(3):569–583

